# Selective inhibition of neuronal Ca_v_3.3 T-type calcium channels by TAT-based channel peptide

**DOI:** 10.1186/s13041-020-00636-y

**Published:** 2020-06-19

**Authors:** Leos Cmarko, Norbert Weiss

**Affiliations:** 1 Institute of Biology and Medical Genetics, First faculty of Medicine, Charles University, Prague, Czech Republic; 2 Institute of Organic Chemistry and Biochemistry, Czech Academy of Sciences, Prague, Czech Republic

**Keywords:** Calcium channel, T-type channel, Ca_v_3.3 channel, TAT-peptide, Inhibitor

## Abstract

Low-voltage-activated Ca_v_3 calcium channels (T-type) play an essential role in the functioning of the nervous system where they support oscillatory activities that relie on several channel molecular determinants that shape their unique gating properties. In a previous study, we documented the important role of the carboxy proximal region in the functioning of Ca_v_3.3 channels. Here, we explore the ability of a TAT-based cell penetrating peptide containing this carboxy proximal region (TAT-C3P) to modulate the activity of Ca_v_3 channels. We show that chronic application of TAT-C3P on tsA-201 cells expressing Ca_v_3 channels selectively inhibits Ca_v_3.3 channels without affecting Ca_v_3.1 and Ca_v_3.2 channels. Therefore, the TAT-C3P peptide described in this study represents a new tool to address the specific physiological role of Ca_v_3.3 channels, and to potentially enhance our understanding of Ca_v_3.3 in disease.

## Main text

Low-voltage-activated Ca_v_3 channels that generate T-type currents display unique biophysical properties that allow them to operate near the resting membrane potential of nerve cells where they generate low-threshold calcium spikes leading to burst firing of action potentials and oscillatory discharges. They play an essential role in shaping the electrophysiological properties of thalamic, olivary, and cerebellar neurons, and alteration of Ca_v_3 channel activity is associated with a number of human neuronal disorders [1, 2]. However, the identification of specific physiological roles associated with each Ca_v_3 isoforms (Ca_v_3.1, Ca_v_3.2, and Ca_v_3.3) is often hampered for several reasons. First, Ca_v_3 channels are often coexpressed in nerve cells. Second, they present a similar electrophysiological signature which renders their molecular identification problematic in native neuronal systems. And third, selective pharmacological tools are not available. Therefore, there is a need for isoform-specific modulators of Ca_v_3 channels in order to better explore their respective physiological functions.

Structure-function studies have identified several channel molecular determinants that are responsible for shaping the unique gating properties of Ca_v_3 channels [3–8]. Recently, we reported the importance of the carboxy terminal domain and showed that the proximal region that is highly conserved across the three Ca_v_3 channel isoforms is essential for the functioning of Ca_v_3.3 channels [9]. The question then arises as to whether an exogenous peptide corresponding to this proximal carboxy terminal region could potentially modulate the expression of Ca_v_3 channels.

**Fig. 1:**
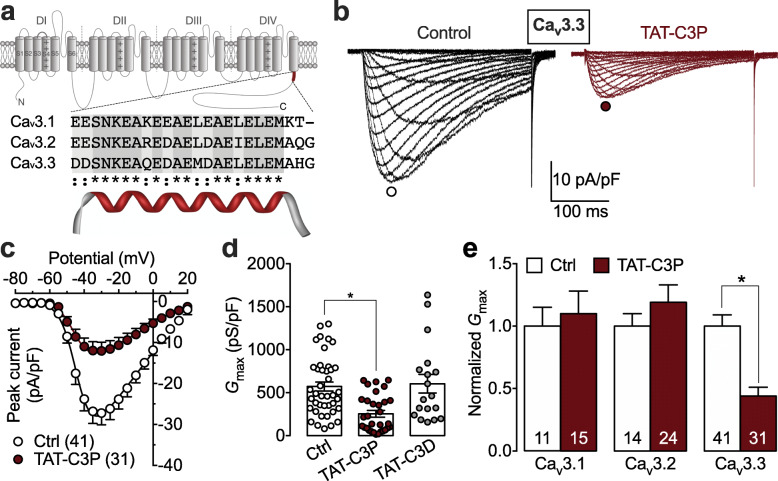
Effect of TAT-C3P peptide on Ca_v_3 channels. **a** Schematic representation of the membrane topology of Ca_v_3 channels and protein sequence alignment showing the conserved proximal region of the carboxy terminus across all three Ca_v_3 channel isoforms (Ca_v_3.1, Ca_v_3.2, and Ca_v_3.3). Molecular modeling indicates that this region may adopt a helical conformation. **b** Representative T-type current traces recorded from cells expressing Ca_v_3.3 channels in response to 300 ms depolarizing steps to values ranging from − 80 mV to + 20 mV from a holding potential of − 100 mV for control (black traces) and TAT-C3P-treated cells (red traces). **c** Corresponding mean peak current density-voltage (*I*/*V*) relationship. **d** Corresponding mean maximal macroscopic conductance (*G*_max_) values obtained from the fit of the *I*/*V* curves with a modified Boltzmann equation for control (white dots), and cells treated with either TAT-C3P (red dots) or the control TAT-C3D peptide (grey dots). **e** Mean normalized *G*_max_ for cells expressing Ca_v_3.1, Ca_v_3.2, and Ca_v_3.3 channels in response to TAT-C3P treatment

To address this issue, we tested the effect of a TAT-based cell penetrating peptide containing the conserved carboxy proximal region of Ca_v_3.2 (TAT-C3P) on recombinant Ca_v_3 channels expressed in tsA-201 cells. Molecular modeling using Phyres^2^ [10] predicted that this peptide may adopt a helical conformation (Fig. 1a). Cells were transfected with 5 μg of cDNA encoding for Ca_v_3.1, Ca_v_3.2, or Ca_v_3.3 channels. Twelve hours after transfection, cells were treated with 10 μg / mL of TAT-C3P peptide (GenScript), or with a control peptide containing a non-conserved distal region of the carboxy terminus of Ca_v_3.2 (TAT-C3D). The effect of the TAT peptide on T-type currents was assessed 48 h later in the whole cell configuration of the patch clamp technique. We observed that treatment of cell with the TAT-C3P produced a potent decrease of the T-type current in cells expressing Ca_v_3.3 channels (Fig. 1b). For instance, in response to a depolarizing pulse to − 30 mV, a 2.3-fold decrease (*p* < 0.0001) in the mean peak T-type current density was observed in cells treated with TAT-C3P (− 12.1 ± 2.1 pA/pF, *n* = 31) compared to control (non-treated) cells (− 27.2 ± 2.9 pA/pF, *n* = 41) (Fig. 1c). The mean maximal slope conductance (*G*_max_) was decreased by 56% (*p* < 0.0001) from 572 ± 53 pS/pF to 254 ± 39 pS/pF (Fig. 1d). This effect was not observed when cells were treated with the control TAT-C3D peptide indicating that TAT-C3P-induced inhibition of Ca_v_3.3 was specifically mediated by the carboxy proximal peptide and not from a non-specific effect that could have resulted from TAT itself (Fig. 1d). Inhibition of T-type currents by TAT-C3P was not associated with additional alteration of the voltage-dependence of activation and inactivation, nor of the recovery from inactivation, and only a slight acceleration of the inactivation kinetics of Ca_v_3.3 currents at hyperpolarized potentials was observed (supplemental Fig. S1). Furthermore, this inhibition was not observed when TAT-C3P was acutely infused into the cells via the patch pipette (supplemental Fig. S2) suggesting that TAT-C3P-induced inhibition of Ca_v_3.3 is likely to have occurred via a regulatory signaling pathway controlling the expression of the channel rather than via direct alteration of the channel activity itself. Finally, we did not observe any significant effect of TAT-C3P on cells expressing Ca_v_3.1 and Ca_v_3.2 channels indicating that this peptide is selective for Ca_v_3.3 channels (Fig. 1e and supplemental Fig. S3). Considering that the proximal carboxy terminal region of Ca_v_3 channels is highly conserved across the three channel isoforms, the observation that TAT-C3P was effective only on Ca_v_3.3 channels suggests the existence of a distinct regulatory mechanism specific for Ca_v_3.3 that may be compromised by the peptide. Additional analysis will elucidate the detailed mechanisms underlying the effect of this peptide.

While several pan Ca_v_3 channel blockers have been described, there is to date no molecule selective for one particular Ca_v_3 isoform [11]. This lack of selective pharmacopeia not only hampered the identification of specific physiological roles for Ca_v_3 channels that in the absence of selective modulator requires the use of genetic or antisense nucleotide approaches, but also compromised the therapeutic development of Ca_v_3 channel modulators. Here, we reported the first non-genetic molecular tool to selectively inhibit Ca_v_3.3 channels in cells, and possibly in vivo. Although the molecular mechanism by which TAT-C3P inhibits Ca_v_3.3 channels remains to be explored in detail, the observation that discreet channel molecular determinants can be harnessed to selectively target a particular channel isoform represents an appealing strategy to study specific physiological functions, and to potentially enhance our understanding of Ca_v_3 channels in disease.

## Supplementary information

**Additional file 1.** Extended methodology and supplemental data.

## Data Availability

All data generated or analyzed during this study are included in this published article and its supplementary information files.

## Abbreviations

TAT-C3P: TAT-based peptide containing the carboxy proximal region of Ca_v_3 channels
TAT-C3D: TAT-based peptide containing the carboxy distal region of Ca_v_3 channels
