# Supplementary material for: Selective inhibition of neuronal Cav3.3 T-type calcium channels by TAT-based channel peptide
Source: Mol Brain. 2020 Jun 19;13:95. doi: 10.1186/s13041-020-00636-y (PMC7304182; doi:10.1186/s13041-020-00636-y)
Supplement: Supplementary file 1 — Additional file 1. Extended methodology and supplemental data. [file 13041_2020_636_MOESM1_ESM.docx]

**Selective inhibition of neuronal Ca_v_3.3 T-type calcium channels by TAT-based channel peptide**

Leos Cmarko^1,2^, Norbert Weiss^1,2,*^

^1^Institute of Biology and Medical Genetics, First faculty of Medicine, Charles University, Prague, Czech Republic; ^2^Institute of Organic Chemistry and Biochemistry, Czech Academy of Sciences, Prague, Czech Republic.

**Additional information**

**Additional methods**

***Cell culture and heterologous expression***

Human embryonic kidney tsA-201 cells were grown in DMEM medium supplemented with 10% fetal bovine serum and 1% penicillin/streptomycin (all media purchased from Invitrogen) and maintained under standard conditions at 37^o^C in a humidified atmosphere containing 5% CO_2_. Heterologous expression of Ca_v_3 channels was performed by transfecting cells with 5 μg plasmid cDNAs encoding human Ca_v_3.1, Ca_v_3.2, and Ca_v_3.3 channel variants using the calcium/phosphate method.

***Peptide synthesis***

TAT-based cell-penetrating peptides were synthesized by GenScript^®^. The TAT-based peptide containing the conserved proximal carboxy terminal region of Ca_v_3.2 channels had the following sequence: *GRKKRRQRRRPQ*EESNKEAREDAELDAEIELEMAQG (TAT-C3P). The control peptide containing a variable distal region of Ca_v_3.2 channels had the following sequence: *GRKKRRQRRRP*QAVSSPARSGEPLHALSPRGTARSP (TAT-C3D). Peptides were dissolved in distilled water and applied onto cells at a final concentration of 10 μg / mL in the culture medium for 48h. For experiments where the peptide was directly infused into the cell via the patch pipette, the peptide was dissolved in the pipette medium at a final concentration of 10 μg / mL.

***Patch-clamp electrophysiology***

Patch clamp recordings of T-type currents in tsA-201 cells expressing Ca_v_3 channels were performed in the whole-cell configuration at room temperature (22-24^o^C). The bath solution contained (in millimolar): 5 BaCl2, 5 KCl, 1 MgCl2, 128 NaCl, 10 TEA-Cl, 10 D-glucose, 10 4-(2-hydroxyethyl)-1-piperazineethanesulfonic acid (HEPES) (pH 7.2 with NaOH). Patch pipettes were filled with a solution containing (in millimolar): 110 CsCl, 3 Mg-ATP, 0.5 Na-GTP, 2.5 MgCl2, 5 D-glucose, 10 EGTA, and 10 HEPES (pH 7.4 with CsOH), and had a resistance of 2–4 MΩ. Recordings were performed using an Axopatch 200B amplifier (Axon Instruments) and acquisition and analysis were performed using pClamp 10 and Clampfit 10 software, respectively (Axon Instruments). The linear leak component of the current was corrected online and current traces were digitized at 10 kHz and filtered at 2 kHz. The voltage dependence of activation of Ca_v_3 channels was determined by measuring the peak T-type current amplitude in response to 150 ms depolarizing steps (300 ms for Ca_v_3.3 channels) to various potentials applied every 10 s from a holding membrane potential of -100 mV. The current-voltage relationship (I/V) curve was fitted with the following modified Boltzmann equation (1):

$$\left( 1 \right) I\left( V \right)= Gmax \frac{(V-Vrev)}{1+ \exp\frac{(V0.5-V)}{k}}$$

with *I*(*V*) being the peak current amplitude at the command potential *V*, *G*max the maximum conductance, *V*rev the reversal potential, *V*_0.5_ the half-activation potential, and *k* the slope factor. The voltage dependence of the whole-cell Ba^2+^ conductance was calculated using the following modified Boltzmann equation (2):

$$\left( 2 \right) G\left( V \right)= \frac{Gmax}{1+ \exp\frac{(V0.5-V)}{k}}$$

with *G*(*V*) being the Ba^2+^ conductance at the command potential *V*.

The voltage dependence of the steady-state inactivation of Ca_v_3 channels was determined by measuring the peak T-type current amplitude in response to a 150 ms depolarizing step to -20 mV applied after a 5 s-long conditioning prepulse ranging from -120 mV to -30 mV. The current amplitude obtained during each test pulse was normalized to the maximal current amplitude and plotted as a function of the prepulse potential. The voltage dependence of the steady-state inactivation was fitted with the following two-state Boltzmann function (3):

$$\left( 3 \right) I\left( V \right)= \frac{Imax}{1+ \exp\frac{(V-V0.5)}{k}}$$

with *I*_max_ corresponding to the maximal peak current amplitude and *V*_0.5_ to the half-inactivation voltage.

The recovery from inactivation was assessed using a double-pulse protocol from a holding potential of -100 mV. The cell membrane was depolarized for 2 s at 0 mV (inactivating prepulse) to ensure complete inactivation of the channel, and then to -20 mV for 150 ms (test pulse) after an increasing time period (interpulse) ranging between 0.1 ms and 7 s at -100 mV. The peak current from the test pulse was plotted as a ratio of the maximum prepulse current versus interpulse interval. The data were fitted with the following single-exponential function (4):

$$\left( 4 \right) \frac{I}{Imax}=A \times(1- exp\frac{-t}{\tau})$$

where τ is the time constant for channel recovery from inactivation.

***Statistical analysis***

Data values are presented as mean ± S>E>M for *n* measurements. Statistical analysis was performed using GraphPad Prism 7. For datasets passing the D’Agostino & Person omnibus normality test, statistical significance was determine using a Student’s t-test. Datasets were considered significantly different for p ≤ 0.05 *.

**Additional figures**

**
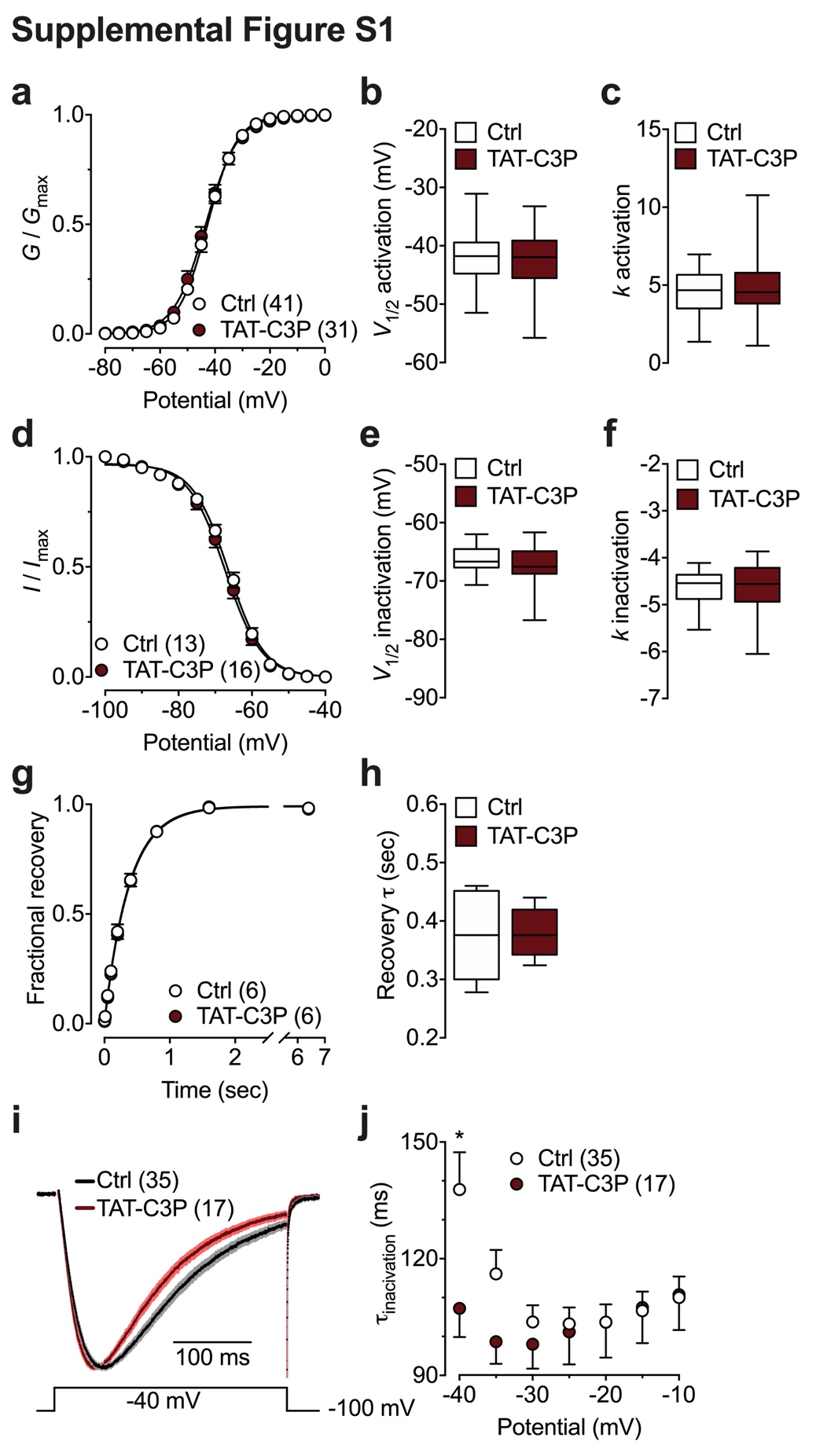
**

**Fig. S1** Effect of TAT-C3P on the electrophysiological properties of Ca_v_3.3 channels. **a** Mean normalized voltage-dependence of Ca_v_3.3 channels in control (white circles) and TAT-C3P-treated cells (red circles). **b** Corresponding mean half-activation potential values obtained from the fit of the activation curves with a modified Boltzmann equation. **c** Corresponding mean slop factor (*k*) of activation. **d-e** Legend same as for (a-c) but for the voltage-dependence of steady state inactivation. **g** Mean normalized recovery from inactivation kinetics. **h** Corresponding mean time constant values of recovery from inactivation obtained from the fit of the recovery curves with a single-exponential function. **i** Mean normalized Ca_v_3.3 current traces recorded in response to a depolarizing step to -40 mV from a holding potential of -100 mV. **j** Corresponding mean time constant values of inactivation obtained from the fit of the decay phase of the T-type current with a single-exponential function.


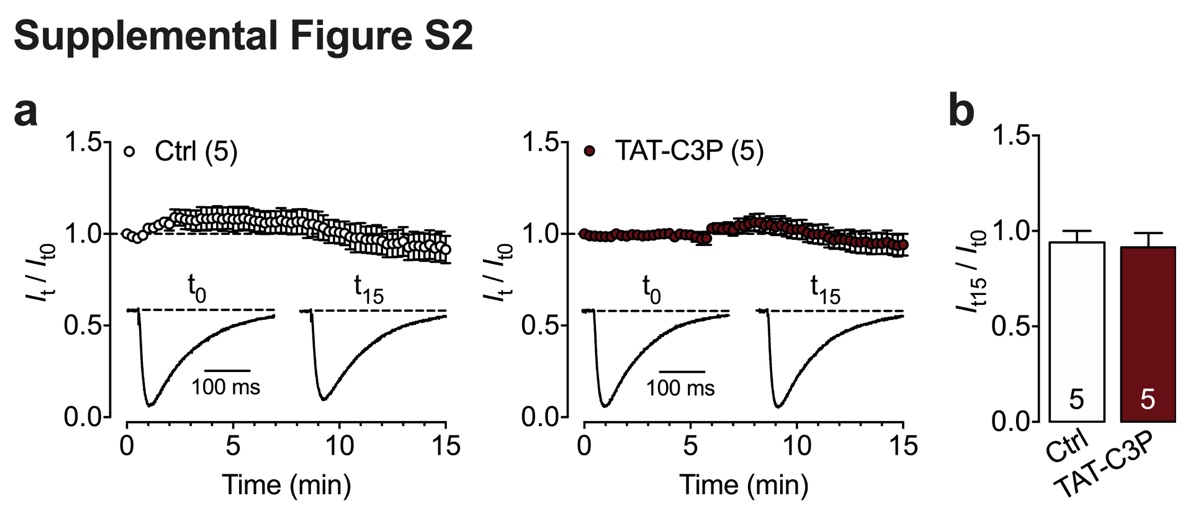


**Fig. S2** Effect of acute infusion of TAT-C3P on Ca_v_3.3 channels. **a** Mean normalized peak T-type currents recording in control cells (white circles) and in cells infused with TAT-C3P via the patch pipette (red circles). Insets represent T-type currents recorded immediately after obtaining the whole cell configuration (t_0_) and after 15 min dialysis (t_15_). **b** Corresponding mean *I*_t15_ / *I*_t0_ values showing that acute infusion of TAT-C3P had no effect on the T-type current.

**
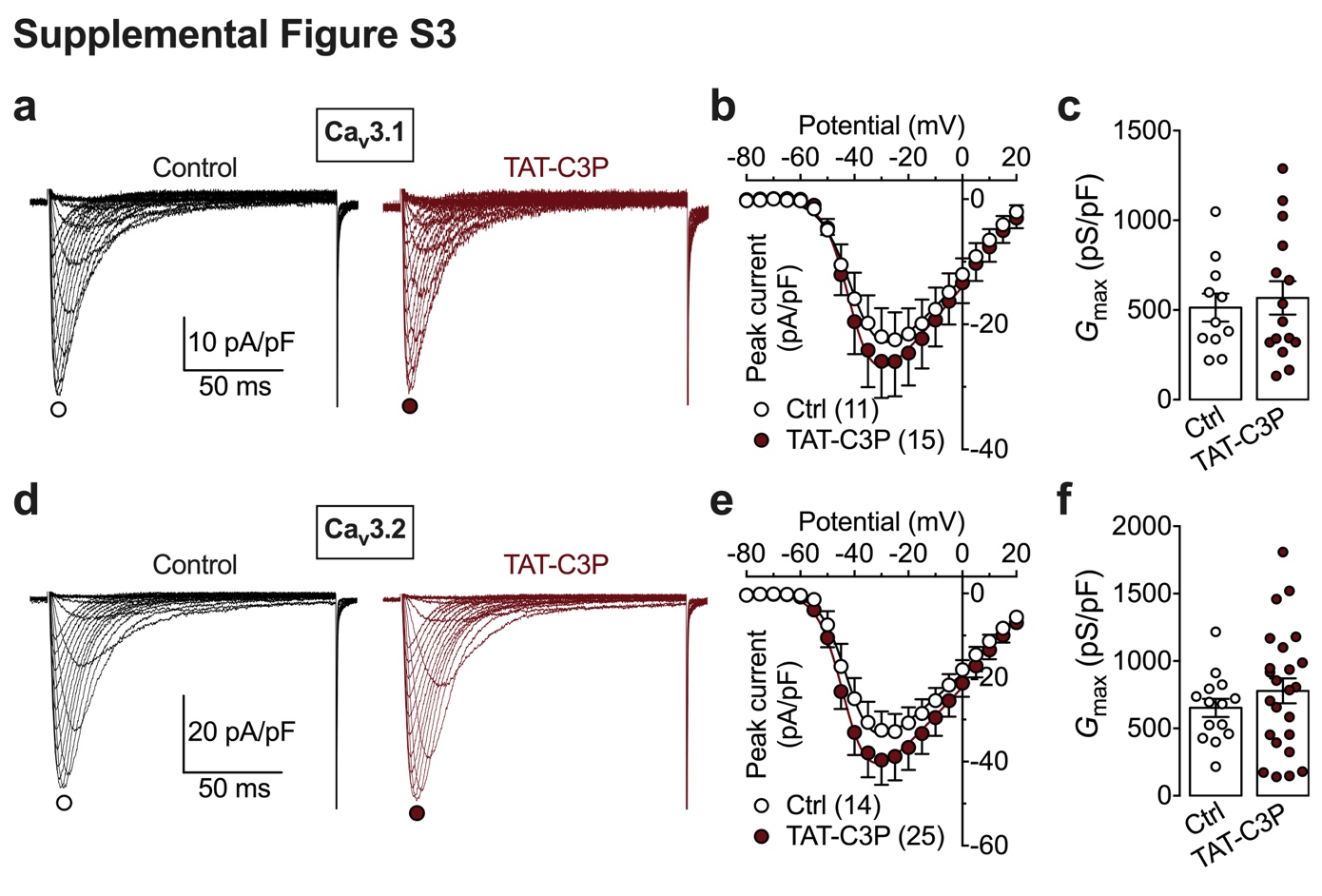
**

**Fig. S3** Effect of TAT-C3P on Ca_v_3.1 and Ca_v_3.2 channels. **a** Representative T-type current traces recorded from cells expressing Ca_v_3.2 channels in response to 150 ms depolarizing steps to values ranging from -80 mV to +20 mV from a holding potential of -100 mV for control (black traces) and TAT-C3P-treated cells (red traces). **b** Corresponding mean peak current density-voltage (*I*/*V*) relationship. **c** Corresponding mean maximal macroscopic conductance (*G*_max_) values obtained from the fit of the *I*/*V* curves with a modified Boltzmann equation for control (white dots), and cells treated with TAT-C3P (red dots). **d-e** Legend same as for (a-c) but for cells expressing Ca_v_3.2 channels.
